# 3D-printed biological cell phantom for testing 3D quantitative phase imaging systems

**DOI:** 10.1038/s41598-019-55330-4

**Published:** 2019-12-11

**Authors:** Michał Ziemczonok, Arkadiusz Kuś, Piotr Wasylczyk, Małgorzata Kujawińska

**Affiliations:** 1 Warsaw University of Technology, Institute of Micromechanics and Photonics, Św. A. Boboli 8 St., 02-525 Warsaw, Poland; 2 Photonic Nanostructure Facility, Faculty of Physics, University of Warsaw, Pasteura 5, 02-093 Warsaw, Poland

**Keywords:** Lithography, Phase-contrast microscopy

## Abstract

As the 3D quantitative phase imaging (QPI) methods mature, their further development calls for reliable tools and methods to characterize and compare their metrological parameters. We use refractive index engineering during two-photon laser photolithography to fabricate a life-scale phantom of a biological cell with internal structures that mimic optical and structural properties of mammalian cells. After verification with a number of reference techniques, the phantom is used to characterize the performance of a limited-angle holographic tomography microscope.

## Introduction

The possibility to observe the phase of transparent, weakly-scattering biological objects^1^ set one of the milestones in both microscopy and biology^2^. A few decades after the introduction of the phase contrast microscopy there is a wide range of phase contrast methods under an umbrella term of quantitative phase imaging (QPI). The technique is no longer a mere observation of phase represented with amplitude - it is now possible to measure the optical path length changes^3^ and retrieve the 3D refractive index (RI) values^4–10^ within biological cells - rapidly and label-free. In many cases the basic 2D QPI techniques have been improved to give access to the three-dimensional RI distribution: thus the digital holographic microscopy (DHM) was extended to become holographic tomography^4^, spatial light interference microscopy^11^ to spatial light interference tomography^12^ or Fourier ptychography^13^ now delivering tomographic measurements^14^. QPI systems have been recently used to study the biophysical properties of biological microstructures such as single cells, cell aggregates and tissues^15–21^. At the same time commercial devices using either spatial light interference (Phi Optics, Inc.^22^) or holographic tomography (NanoLive, Ltd^23^ and TomoCube, Inc.^24^) entered the market, readily tailored for microbiological applications. Despite the commercialization of these QPI systems, their performance have not been fully quantified experimentally^25^. The metrological and comparative stage is especially important in the case of the techniques that intrinsically exhibit anisotropic spatial resolution. One example is limited-angle holographic tomography (LAHT)^26,27^ as compared to its version based on the object rotation with illumination scanning^28^, in which additional reconstruction artifacts appear. In contrast, in the case of other well-established 3D imaging techniques the importance of comprehensive and systematic approach to the calibration of the measurement system has already been recognized^29–32^. New calibration targets, tailored to specific applications have been developed^33–36^ for comparative studies based on data from various system configurations and numerical procedures. For the QPI techniques, however, the most advanced test targets so far have been purely numerical^37^, while verification of experimental results was based on unknown^38–42^ or very simple objects - e.g. uniform micro-spheres^25,43,44^. This approach is far from optimum and unable to recreate the challenges present during the measurements of biological specimens, in which the reference data for the geometry and RI is missing.

In this paper we demonstrate the design and fabrication of a micrometer scale phantoms with optical and structural properties and features typically found in mammalian cells. The phantom is manufactured by 3D laser photolithography (direct laser writing) with the RI engineered during a single fabrication step. The phantom is characterized with a number of reference techniques - scanning electron microscopy and white light interferometry for the geometry and isotropic holographic tomography for the refractive index distribution. Further, it application in testing a QPI instrument - a limited-angle holographic tomography microscope - is presented.

## The Cell Phantom Design

The key to the design of a 3D phase microscopy phantom is the comprehensive understanding of the characteristics of a typical object under study that influence its interaction with light. Secondary criteria (such as the shape, feature size and the range of RI values) result from the targeted technique or system and the intended application which ultimately determine the usability of such calibration object. All of the above have to be within reach of the available manufacturing techniques and accessible for verification with other modalities.

QPI methods are based on retrieving the optical path length differences introduced by the specimen with respect to its surrounding medium and therefore require phantoms with precisely engineered spatial distribution of the refractive index. When biological cells are imaged with 3D QPI techniques, the sub-cellular structures of interest are often the nucleolus and lipid droplets with the RI difference relative to the cytoplasm of the order of 0.01 and 0.05, respectively^16,45,46^. Along with the shape and dimensions of a typical adherent cell, these features are the basis for the design of our cell phantom. For the comprehensive metrological evaluation of a 3D QPI technique, the test structure should also accommodate targets for the resolution quantification (defined as the minimum distance between two distinguishable points). The theoretical resolution varies according to the method^5,39^, however it may be expected to be around 300 nm in the lateral and 700 nm in the axial direction with the RI accuracy around 0.001.

**Figure 1:**
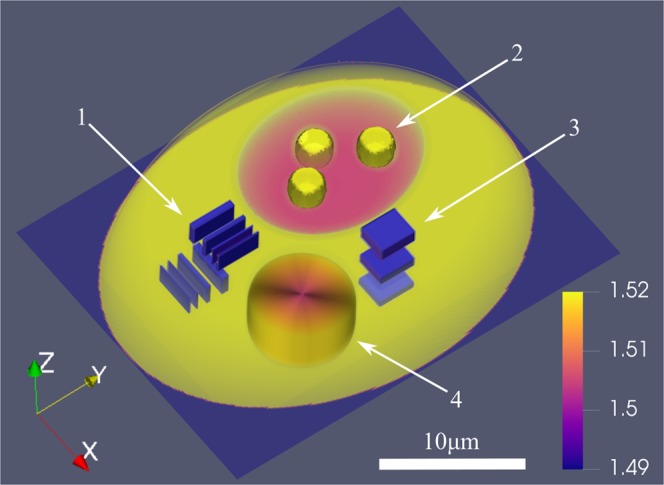
Isometric view of the cell phantom design. Sub-cellular structures are enclosed within truncated ellipsoid with the maximum external dimensions in the XY plane equal 30 × 25 *μ*m and the height of 11 *μ*m. 1 - resolution test in the X and Y directions (line widths: 300, 500 and 700 nm, ΔRI = 0.03); 2 - spherical nucleoli (2.6 *μ*m in diameter) suspended in the low-RI region, representing the nucleus (ΔRI = 0.015); 3 - resolution test in the Z direction (line widths: 800, 1100 and 1400 nm, ΔRI = 0.03); 4 - cylinder with a radial RI gradient (ΔRI = 0.015).

Our biological cell phantom design and its key features are shown in Fig. 1. There are three spherical nucleoli suspended in a region of lower RI (representing the nucleus), which is a common feature in many cells^45,47,48^. The structures are used to verify the ability to retrieve boundaries of large sub-cellular structures and their RI. In the vicinity of the nucleus-like area there are three resolution test targets of high RI difference along the X, Y and Z axis. These also enable us to quantify the errors associated with the presence of high RI gradients^49^, such as lipid droplets^16^. The next feature within the phantom is a cylinder with slow radial gradient of the RI that represents natural RI changes within cells. It is particularly useful for the evaluation of the tomographic reconstruction strategies that may assume piecewise constant RI distribution^50^. The last feature is the thin wall separating the low RI nucleus from the surrounding immersion medium, which is used to determine the ability to retrieve the boundaries of the specimen^51^.

## The Cell Phantom Verification

A reliable calibration object needs its key parameters verified with the accuracy exceeding the performance of the calibrated systems. This usually involves other measurement modalities, as the newly developed measurement techniques lack established calibration protocols.

**Figure 2:**
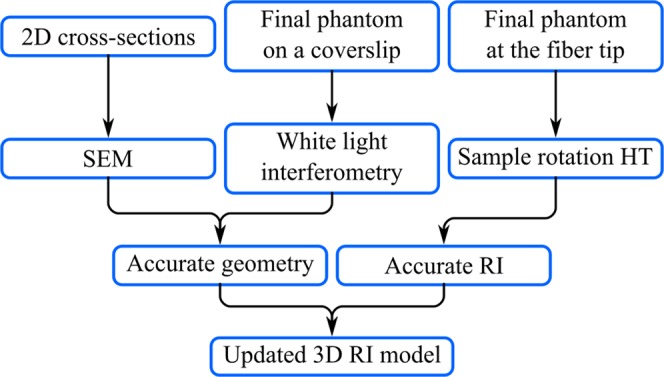
Overview of the phantom fabrication and verification process providing reference geometry and RI data, that leads to the accurate 3D refractive index distribution model.

Verification of the cell phantom requires determination of its 3D geometry and the RI distribution. The dimensions of the phantom features were measured using scanning electron microscopy (SEM) that provides more than an order of magnitude higher spatial resolution and accuracy than the target optical microscopy techniques. However, applicability of SEM to the investigation of the vertical features is limited and thus the height of the phantom is measured with the white light interferometry. For the reference RI distribution it is vital to use a QPI technique that exhibits isotropic resolution - in our case holographic tomography with sample rotation supported by illumination scanning^18,28^ provides sufficient accuracy of the RI reconstruction (of the order of 10^−4^) and was used for measuring the average reference RI values across the phantom printed at the tip of the optical fiber. Updating the phantom design with the data from these measurements provides the closest numerical representation of the physical object for further analysis. The summary of the verification process is shown in Fig. 2.

**Figure 3:**
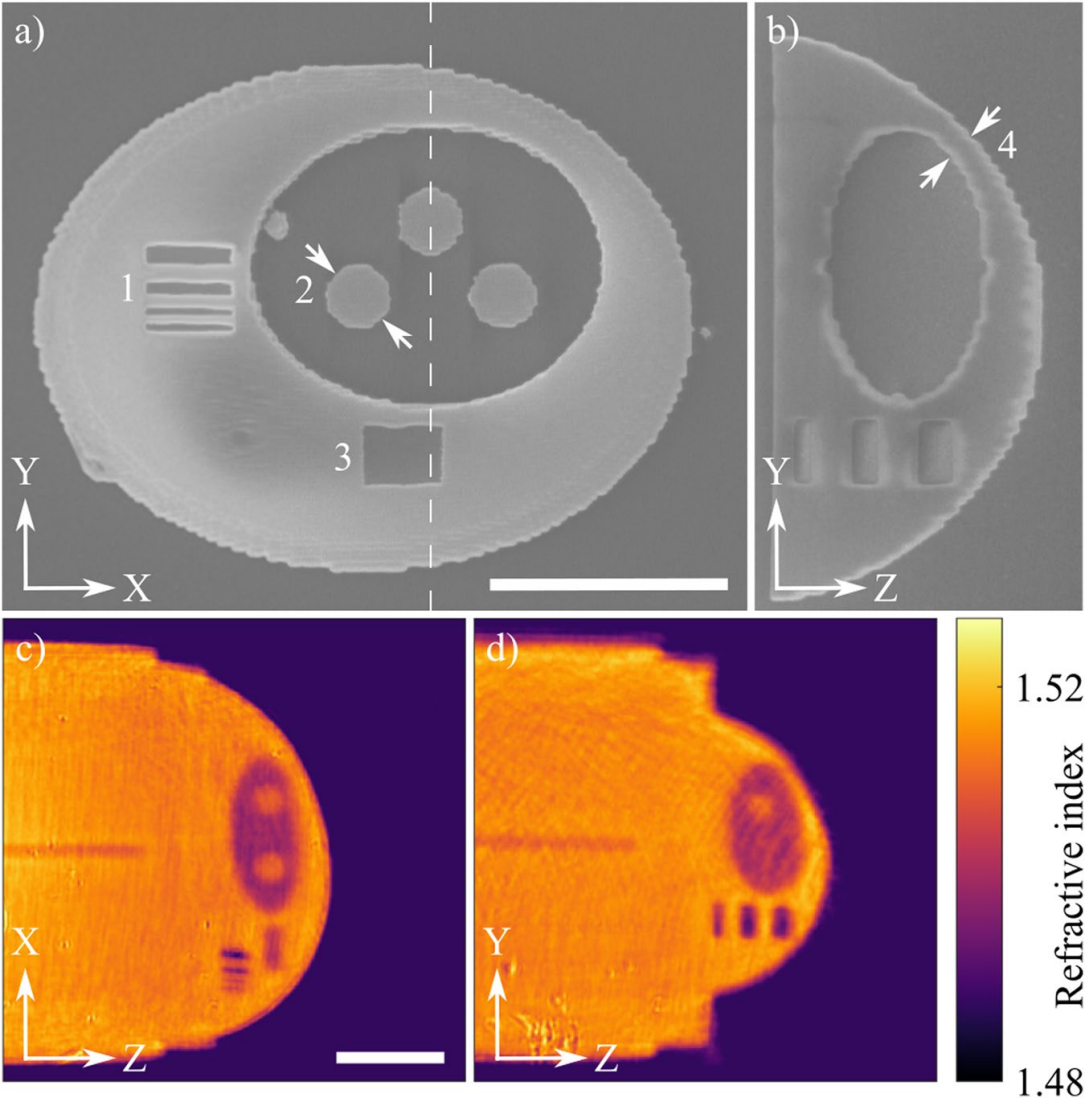
(**a**,**b**) SEM images of the phantom slices. 1 - the resolution test in the Y direction; 2 - the spheres representing the nucleoli; 3 - the resolution test in the Z direction; 4 - a thin wall separating internal structures from the immersion medium. (**c**,**d**) Cross-sections of the 3D RI distribution of the phantom showing final arrangement of the features along the Z axis. Phantom sits on a 3D printed support - 70 *μ*m long cylinder to offset the structure from the surface of the optical fiber (not visible). The scale bars are 10 *μ*m long.

The size of the photolithographic voxel and the critical dimensions of the phantom were obtained using SEM and the standard deviation was calculated based on 10 printed structures. The measured diameter of the voxel at high exposure dose was 340 ± 15 nm, while its height was 1244 ± 36 nm. The width of the lateral resolution test lines was within 30 nm of their designed values in the case of 300 and 500 nm lines and within 60 nm for 700 nm. The relative standard deviation of the widths are well below 5%. The error of the vertical test is below 100 nm with the deviation below 7% of the nominal height. The diameter of the spheres (representing the nucleoli) in the XY plane indicated in Fig. 3a was 2582 ± 59 nm. The minimum thickness of the wall separating the low RI nucleus from the surrounding medium (see Fig. 3b,d) was 1400 ± 86 nm.

Next, the phantom was characterized with holographic tomography. The reconstructions obtained in the isotropic mode demonstrate that the 3D distribution of the RI within the phantom is in agreement with the initial design and the SEM images, as shown in Fig. 3c,d. The mean reference values of the RI, presented in Table 1 along with its standard deviation, have been extracted from regions of interest within the structure.

**Table 1:** Retrieved refractive indices of the polymer with various degrees of polymerization.

Exposure dose	Refractive index
High	1,5167 ± 0,0008
Low	1,5021 ± 0,0012
Unexposed	1,4882 ± 0,0006

## Application of the Cell Phantom to QPI System Testing

**Figure 4:**
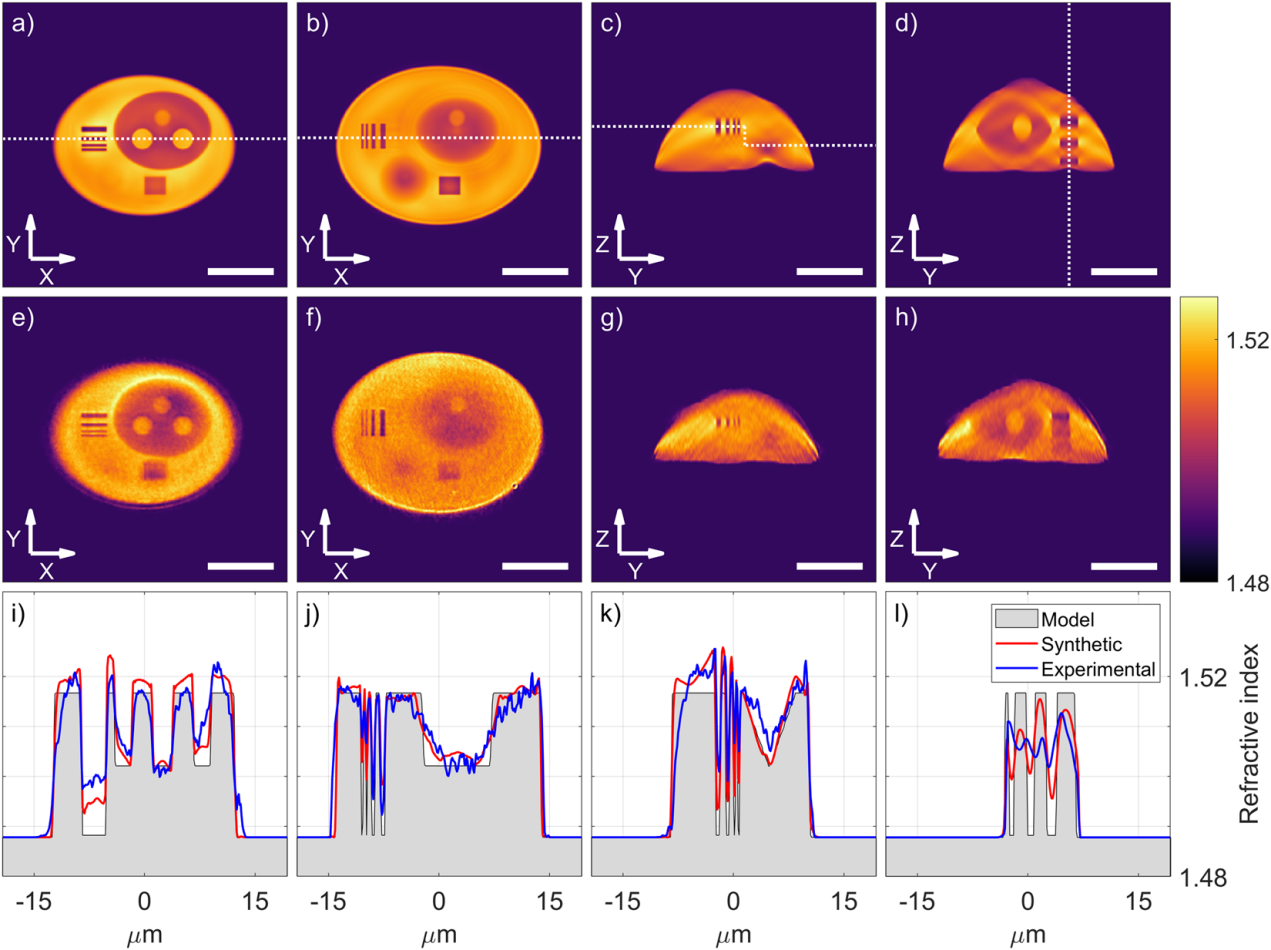
Numerical reconstruction (**a**–**d**) and the experimental results (**e**–**h**) of the 3D refractive index measurements. Cross-sections along the white dotted lines (**i**–**l**) show a comparison between both reconstructions and the model based on the reference geometry and the RI data. The scale bars are 10 *μ*m long.

The first row in Fig. 4 the first row (panels a–d) presents cross-sections of the 3D refractive index distribution of the numerical reconstruction of the phantom, followed by the corresponding cross-sections obtained in the LAHT system (e–h). The RI profiles from both reconstructions and the model are plotted in panels (i–l). Quantification of the reconstruction quality based on selected features is summarized in Table 2.

**Table 2:** The evaluation parameters of the phantom reconstructions and their values in the model, the synthetic and the experimental reconstructions.

	Feature	Model	Synthetic	Experimental
P-V ratio	Lateral 300 nm X	1	0,48	0,17
	Lateral 700 nm X	1	0,62	0,83
	Vertical 800 nm	1	0,34	0,07
	Vertical 1400 nm	1	0,59	0,34
Nucleoli	Lateral size [*μ*m]	2,72	2,72	2,59
	Vertical size [*μ*m]	3,00	3,55	3,62
	Mean RI	1,5167	1,5184	1,5151
	Gradient RI P-V	0,0146	0,0165	0,0136
	Cumulative RI [%]	100	99,4	92,0

External boundaries of the phantom are retrieved very accurately, including in the Z direction - the total height is 11.1 *μ*m, 200 nm less than the value measured with the white light interferometer. The cross-sections along the resolution tests prove that the system is capable of resolving features down to 300 nm with a period of 600 nm in the XY plane, as well as 800 nm features spaced by 2500 nm in the Z direction, however the RI values are not accurate - smaller features exhibit low contrast, thus the visibility of subsequent test lines can be quantified as the P-V ratio obtained from the reconstruction and the model. This effect is observed in both synthetic and experimental reconstruction, therefore it may be caused by insufficient sampling (here (130 nm)^3^), iterative data replenishment routine or the high gradient of the RI not satisfying the requirements of a weakly scattering sample.

The lateral diameter of the nucleoli from both synthetic and experimental data is reconstructed very accurately (with error of 130 nm), while vertically they are stretched by around 20% (elongation artifacts are typical in the limited angle version of holographic tomography). Overestimation of the RI of the spheres in the synthetic reconstruction may originate from accumulation of the RI in the lateral cross-sections near the center of mass of the structure (first column in Fig. 4) and should similarly affect the experimental data. Measured RI of the spheres, however, is underestimated and indicate errors in the experimental data. Cumulative RI indicates possible loss of dry mass during the reconstruction process (approximately 0.5%) and its significant underestimation (8%) in the experiment. The algorithm used for the reconstruction has retrieved the gradient profile very accurately in both reconstructions with the RI P-V error within 0.002 of the designed value.

## Discussion

The presented methodology, involving comparison of the experimental reconstruction with the model and its synthetic reconstruction, provides valuable insight into metrological performance of the system and points out the weaknesses of the algorithms. Distortions of the RI near the structure center of mass, as well as those associated with high gradient of the RI indicate the shortcomings of the reconstruction algorithm, which along with the anisotropic resolution constitute the majority of RI retrieval error. The identified issues in the experimental part of the measurement procedure include the overall underestimation of the RI, further degradation of the reconstruction along the vertical axis (e.g. in the resolution test and missing horizontal boundaries of the nucleus, most likely due to imperfect illumination vectors) and noise.

The laser printed phantom slices investigated with SEM were subject to anisotropic shrinkage and capillary forces during air-drying. The resulting deformation of their shape and wavy contours are visible primarily near the fine details such as the resolution test lines (1 in Fig. 3a) and were a major source of the width difference between the samples. Preventing or compensating for the polymer shrinkage^52^ may significantly reduce the uncertainty of the dimensions and provide a more accurate model. Techniques such as single-anchor support and critical point drying^53,54^ may promote isotropic shrinkage and reduce the stress during rinsing, rendering the fabrication process more accurate and predictable. The aforementioned issues affect the 3D structure to a lesser degree than the individual slices and their overall contribution to the discrepancy between the investigated 2D structures and the 3D phantom was estimated to be below 50 nm in the case of lateral and, due to lower stiffness, 100 nm in the vertical cross-sections. The worst-case error estimation leads to the conclusion that the SEM examination provides the reference data exceeding the theoretical resolution of the QPI methods in each axis by at least a factor of two.

Temporal laser power variations, power density distribution within the voxel and the repeatability of the RI are the major concerns regarding the reliability and repeatability of the phantom fabrication process. They are addressed by the fact that each point within the structure is exposed to the light intensity exceeding the polymerization threshold multiple times, thus averaging the spatial (the voxel size and the hatching distance are comparable to the LAHT resolution) and temporal (delay of seconds and minutes for neighboring lines and layers, respectively) variations of the RI during fabrication. At the same time, the uncured regions of liquid resin are susceptible to uncontrolled polymerization induced by defocused/scattered printing beam, ambient light or illumination beam during optical measurements. As for the fabrication, any influence have already been accounted for with the identical printing conditions for all samples and no measurable change of the RI related to the overexposure was visible during further analysis. Additionally, polymers are known for their instability and degradation - we have determined, that over the course of 3 months the RI change in the printed phantom was below 10^−3^, however more data is needed to meet the reliability and long-term stability requirements for the calibration structure.

## Conclusions

In this work we have developed a methodology for the design, fabrication, verification and utilization of a biological cell phantom to visualize and quantify metrological performance of QPI systems such as holographic tomography with illumination scanning. Considering a variety of hardware and software solutions for QPI techniques in general, such phantoms are critical components for the development of measurement systems - either commercial or research. Moreover, reliable experimental data is crucial for advancement of reconstruction strategies beyond single scattering approximation, such as machine learning^55^ or multiple scattering approach^56^. Our works deliver a phantom reproducing features found in biological samples at the sufficient level of accuracy for both the geometrical shape and refractive index distribution, as well as provides scalability and versatility to accommodate a number of QPI instruments and reference techniques. However, more data is required regarding dispersion of the polymers, as well as their stability and the reproducibility of the phantom.

Finally, we have also demonstrated the feasibility of achieving high resolution and high contrast of 3D RI distribution in a single fabrication step as well as the verification feedback loop enabled by SEM and sample rotation holographic tomography that may find applications to other fields, such as design and optimization of complex micro-optical components.

## Methods

### Fabrication

We use three dimensional two-photon photolithography (TPP, also known as direct laser writing), in which two-photon absorption leads to curing (solidification) of a liquid polymer in a confined volume around the focus of a femtosecond-pulsed laser beam. Scanning the focal region along a given trajectory leads to formation of an arbitrary, phase-only 3D structures^57^. All structures presented in this work were fabricated with Photonic Professional GT workstation (Nanoscribe GmbH) with a piezo scanning stage and a 100x microscope objective having numerical aperture (NA) of 1.4. The minimum solidified volume (voxel) size is 100 nm in the lateral and 500 nm in the axial directions^58^, positioned in the 3D space with the accuracy of 10 nm. Polymer used for the cell phantom fabrication (IP-L 780, Nanoscribe GmbH) is commercially available and optimized for TPP in terms of printing resolution and shrinkage. Once the writing process is complete, the unexposed photoresist is removed (rinsed off) in an isopropyl alcohol bath for 20 min and then the structure is blow-dried with air. All structures have been designed and fabricated using the software provided with the workstation - DeScribe 2.5 and NanoWrite 1.8.3.

**Figure 5:**
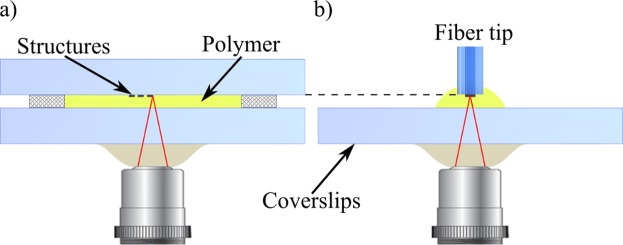
Direct laser writing configuration for printing the structures on (**a**) a coverslip and (**b**) an optical fiber tip. The structures are fabricated layer by layer towards the microscope objective, following the dip-in photolithography principle^63^.

The most desired feature of the phase structures is the well-defined spatial variation of the RI. In the photo-cured polymer, the RI is proportional to the degree of the monomer cross-linking and as such can be controlled via the light exposure dose^59^. Although custom RI distribution can be achieved using many different parameters (such as scan speed, voxel overlap or exposure time), in our experiments we only varied the laser power setting at each voxel. The maximum RI contrast of the solidified polymer available in our setup is of the order of 2 · 10^−2^, which corresponds to the laser power of 20 mW and 13 mW for high and low RI regions, respectively. The RI difference can be increased to 4 · 10^−2^ by leaving regions of uncured, liquid polymer. This RI range is sufficient for fabrication of all the sub-cellular structures within the artificial cell. A number of 3D QPI techniques (especially full-angle or isotropic holographic tomography) require sample rotation to perform the measurement. To use our phantom in these systems, we fabricate it at the tip of an optical fiber^60^, while to ensure compatibility with other 3D QPI methods used for biomedical applications the phantoms are printed on 170 *μ*m coverslips. In order to maintain the same printing conditions all structures are printed on a support, 90 ± 1 *μ*m from the polymer-coverslip interface, as shown in Fig. 5.

### Verification

Examination with SEM provides data on the voxel size for specific fabrication parameters and the dimensions of the phantom, including subcellular structures, based on the developed sample containing sets of lines and relevant cross-sections of the phantom. In order to show the boundaries of the high RI regions in the original phantom, the low RI regions (the nucleus) were left uncured. Measurements along the Z axis were possible by tipping over the vertically printed lines and structures (such as the full XZ cross-section that is only one line thick in the Y direction) flat on the substrate. Then, the fabricated structures were sputter coated with a 25 nm layer of gold (Q150R, Quorum Technologies) and examined with a scanning electron microscope (EVO MA 10, Zeiss).

The next step of the phantom geometry determination (the external shape) is performed with white light interferometry and uses phantom fabricated directly on a coverslip with no additional coating. Reference measurement of the final height profile of the phantom was performed with a commercial white-light interferometer (NT2000, Veeco) on the same sample that was later used in the LAHT experiments.

**Figure 6:**
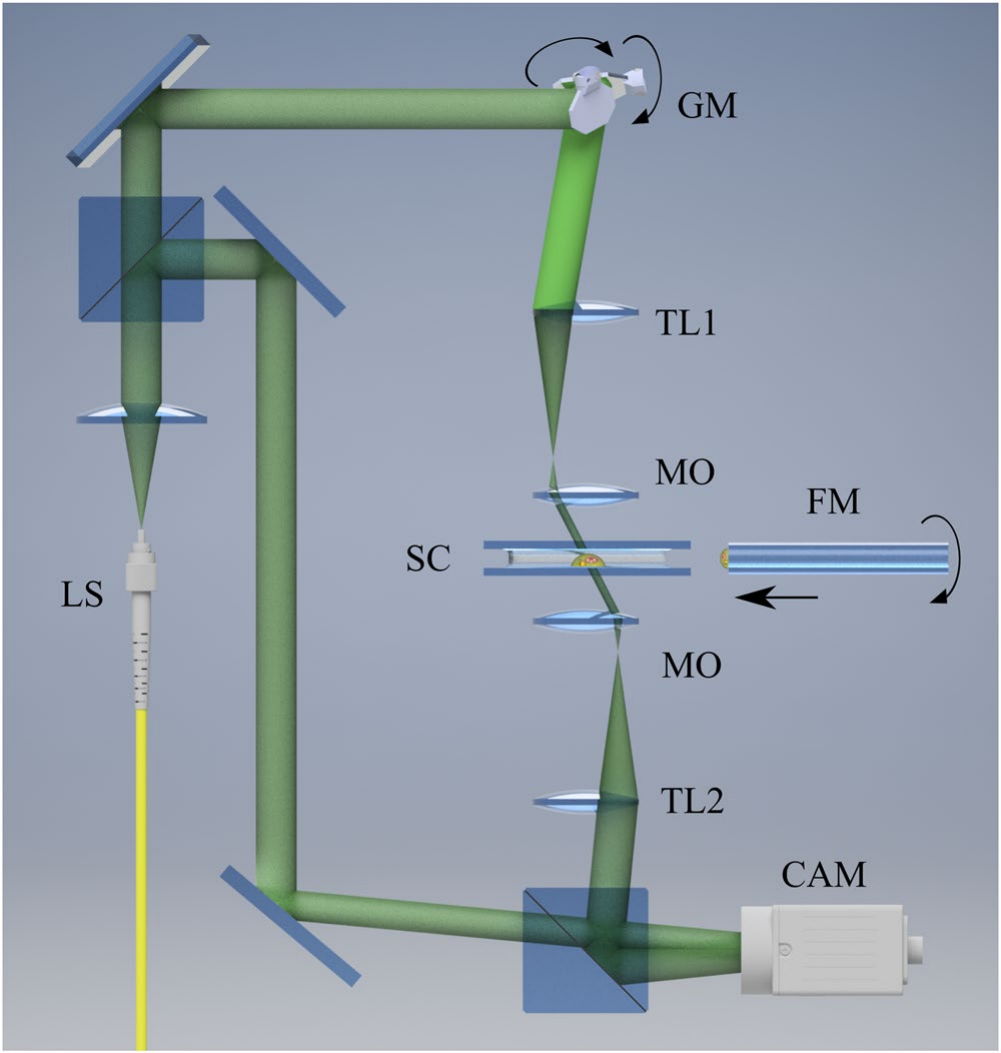
Holographic tomography system used in the experiments: fiber-coupled He-Ne laser (LS, *λ* = 632.8 nm), GM - 2D galvanometer mirrors, TL1, TL2 - tube lenses (${f^{\prime} }_{TL1}=150\,mm$, ${f^{\prime} }_{TL2}=200\,mm$), MO - microscope objective (100x, NA = 1.3 for illumination and 40x, NA = 1.3 for imaging), SC - sample chamber. In the illumination scanning mode the object under study is attached to the bottom coverslip, while in sample rotation (isotropic) mode, an optical fiber with the phantom printed on its end is attached to a motor and introduced between the coverslips from the side (FM - rotating fiber).

The last step of the verification procedure, measuring the 3D RI distribution, was performed using an off-axis Mach-Zehnder digital holographic microscope, working in both sample rotation and illumination scanning mode (Fig. 6) to provide isotropic resolution holographic tomography. The sample was rotated to four angular positions, at which the illumination scanning (limited-angle) holographic tomography measurements was performed. The phantom for the holographic tomography (either on a coverslip or on a fiber tip) was immersed in oil (Cargille-Sacher Laboratories Inc., series A) with the RI of 1.4878 ± 0.0002.

Tomographic reconstruction algorithm used in this work was based on direct implementation of the Fourier diffraction theorem in the Rytov approximation. LAHT data were further processed with the iterative Gerchberg-Papoulis approach^26^ with additional spatial support provided by the 3D mask computed with the total variation iterative constraint method^61^.

### Reconstruction quality assessment metrics

The numerical phantom model is obtained during the verification process and any discrepancies between the model and the experimental reconstruction can be considered as the total error. However, with proper numerical tools one can simulate the measurement process and obtain the corresponding synthetic reconstruction of the model that contains errors associated with the method and the numerical procedures. Through the analysis of the three sets of data (model, numerical reconstruction and experimental reconstruction) it is possible to separate the contributions of the hardware and software components to the total error.

For the quantitative analysis, a full reference image quality assessment metric^62^ is typically used for the 3D data or a 2D cross-sections such as the universal quality index Q, often applied to tomographic reconstruction analysis. Our metrological evaluation is based on quantification of selected features of the phantom: the external dimensions, peak-to-valley (P-V) value of the RI of the resolution test lines, lateral and vertical radius of the nucleoli (determined as the full width at half maximum of their RI profile), as well as their mean RI within corresponding ellipsoid, gradient P-V and cumulative RI (proportional to the cellular dry mass, integrated over the total measurement volume). This set of features provides an objective way to benchmark the metrological performance of the optical measurement system with regards to its applications and deliver meaningful parameters, which along with the qualitative approach may facilitate their further development.

## Data Availability

The datasets generated and/or analyzed during the current study are available from the corresponding author on reasonable request.
